# External Validation of a Novel Landmark-Based Deep Learning Automated Tibial Slope Measurement Algorithm Applied on Short Radiographs Obtained in Patients With ACL Injuries

**DOI:** 10.1177/23259671251333607

**Published:** 2025-05-05

**Authors:** Kyle R. Martin, Sanna Haaland, Andreas Persson, Sung Eun Kim, ByeongYeong Ryu, Jun Woo Nam, Du Huy Ro, Eivind Inderhaug

**Affiliations:** * Department of Orthopaedic Surgery, University of Minnesota, Minneapolis, Minnesota, USA; † Department of Orthopeadic Surgery, Centra Care, Saint Cloud, Minnesota, USA; ‡ Oslo Sports Trauma Research Center, Norwegian School of Sports Sciences, Oslo, Norway; § Sports Traumatology Arthroscopy Research Group, Faculty of Medicine, University of Bergen, Bergen, Hordaland, Norway; ‖ Oslo University Hospital, Oslo, Norway; ¶ Department of Orthopaedic Surgery, Seoul National College of Medicine, Seoul, Republic of Korea; # Connecteve Co, Ltd, Seoul, Republic of Korea; ** Innovative Medical Technology Research Institute, Seoul National University Hospital, Seoul, Republic of Korea; †† Haraldsplass Deaconess Hospital, Bergen, Norway; ‡‡ Haukeland University Hospital, Bergen, Hordaland, Norway; Investigation performed at Haraldsplass Deaconess Hospital, Bergen, Hordaland, Norway

**Keywords:** posterior tibial slope, anterior cruciate ligament, radiographs, automated reading, deep learning, artificial intelligence

## Abstract

**Background:**

Deep learning algorithms can aid medical decision-making by performing routine tasks without any human error. Reading of standardized radiographs lends itself well to a computerized approach. The posterior tibial slope is increasingly recognized as a factor in lower leg biomechanics. Slope readings should, therefore, be readily available when considering knee ligament or knee replacement surgery.

**Purpose/Hypothesis:**

The purpose was to externally validate a deep learning model developed for posterior tibial slope readings by applying an independent data set, not included in initial development, for testing the reliability of the model, compared with human performance testing. It was hypothesized that a computerized approach would yield a reliability similar to that of human analyses.

**Study Design:**

Descriptive laboratory study.

**Methods:**

A consecutive series of lateral knee radiographs obtained in patients undergoing anterior cruciate ligament surgery were eligible for inclusion. Two independent experienced clinicians individually assessed the tibial slope measurement to establish the interreader reliability. Furthermore, all images were processed by the newly developed model for the automated readings. Intrarater and interrater reliability were thereafter established between readers and between manual and automated readings, measured by intraclass correlation coefficients (ICCs). Time consumption between methods was noted. Extreme differences between the 2 methods were analyzed for potential errors.

**Results:**

A total of 289 radiographs were included in the study and therefore analyzed by both the manual and the automated method. A mean tibial slope of 9.7° (SD, 2.7°; range, 3.0°-19.1°) was found. The interrater and intrarater measurements between the independent measurers for the 2-circle method were 0.86 and 0.92. Furthermore, the intrarater agreement of the model was 1.00, while an ICC between 0.73 and 0.80 was found when comparing automated with manual measurement. The mean time consumption for manual readings was 52.5 seconds, while for automated readings it was 28.2 seconds.

**Conclusion:**

In this external validation of a newly developed model for automated readings of tibial slope measures, a perfect intrarater reliability and a good interrater reliability were seen. Although the model needs further refinement in reporting the tibial slope as compared with a gold standard manual measurement, it clearly demonstrates the elimination of human variance with repeat readings and less time consumption than that with human effort.

The posterior tibial slope (PTS), defined as the inclination of the tibial plateau from anterior to posterior, has been identified as a crucial factor affecting forces acting through a weightbearing knee.^1,4,6,7^ Recent studies have suggested a relation between an increased PTS and the risk of anterior cruciate ligament (ACL) injuries, as well as a risk of failure after ACL reconstruction (ACLR).^7,12,14^ In the setting of a high PTS, it has been proposed that a slope-reducing osteotomy should be considered to reduce the risk of failed surgery and new injuries.^17^ Furthermore, the tibial slope has also been found to be of importance in knee arthroplasty.^17^ An aberrant slope has been related to excessive loosening of implants due to the force distribution acting on the joint. For these reasons, the PTS has become an increasingly important factor to consider as part of the surgical decision-making process for common knee procedures such as ACLR and total knee arthroplasty.^16^

Applying algorithms founded in artificial intelligence to standardized medical procedures can save time, reduce human error, and help one make unbiased decisions.^8^ Moreover, such algorithms could enable scarce human resources to be allocated to other more pressing tasks. Interpretation of plain radiographs, including the evaluation of PTS, is an example of a task that lends itself well for such integration of artificial intelligence in clinical practice. In addition to potentially reducing the human variability observed in repeat manual measurement of the same images, an automated method of PTS calculation could serve as a standard reference that aids any future comparison across patient populations. At present, there are a plethora of PTS measurement techniques,^13^ and no clear consensus regarding which method provides the best representation of the true bony morphology of the proximal tibia. An efficient and valid deep learning algorithm would, therefore, be a valuable addition to the profession in creating a more uniform description and communication of the PTS.

An automated PTS measurement platform was created and internally validated based on radiographs obtained in patients from the Republic of Korea. In the index study, the automated PTS was validated using a novel landmark-based method that was both accurate and efficient when compared with manual measurement. The purpose of this study was to test the accuracy and efficiency of the PTS model, relative to manual human measurement, when presented with lateral knee radiographs obtained from patients from Norway with an ACL deficiency. This external validation is a crucial step in ensuring the generalizability and reliability of the model when evaluating radiographs obtained in a different patient cohort than was used for the initial model development and testing. The hypothesis of this study was that the model would demonstrate a similar level of accuracy and superior efficiency to manual PTS measurement when applied to the Norwegian radiographs.

## Methods

### Ethics

The project was presented for and approved by the Regional Ethical Committee (Helse Vest ID 2023:656877) in Norway. The original model development similarly received institutional review board approval in the Republic of Korea (No. 2100-200-1269). Informed consent was waived in both countries due to the retrospective design of the study, and no patient data were transferred between institutions.

### Index Model Development

**Figure 1.**
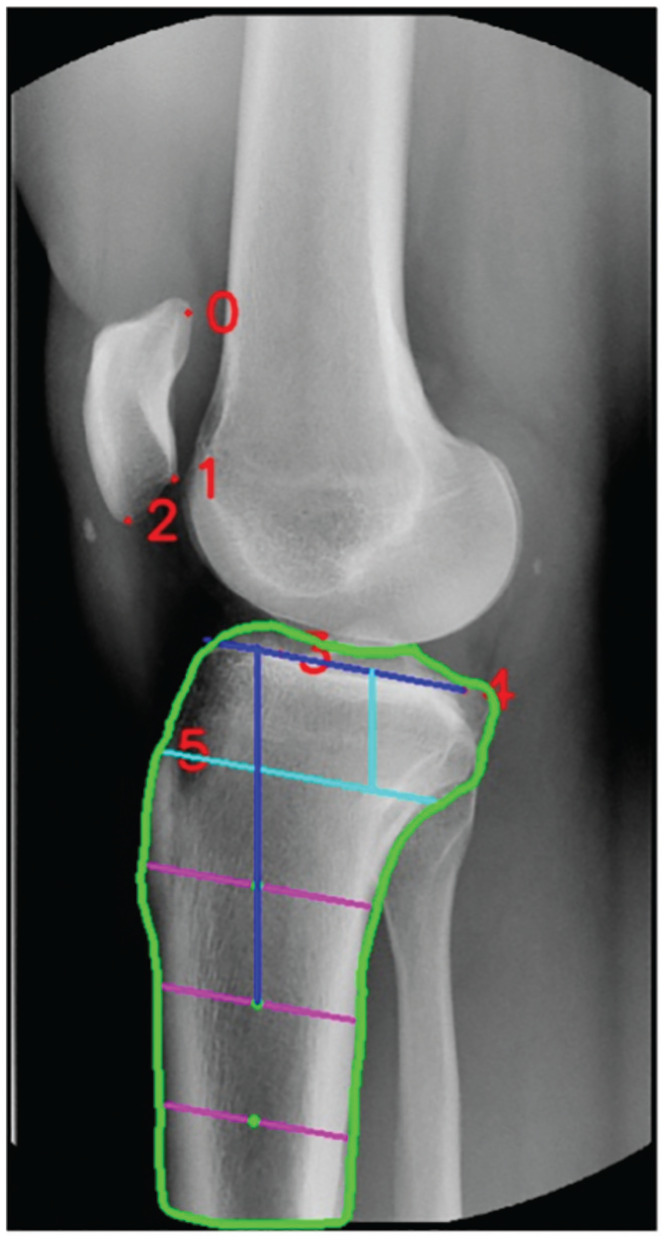
Automated measurement of the posterior tibial slope on a short lateral knee radiograph using the 2A/3A method, demonstrating a slope of 11°. The green outline represents the automated identification of the tibial cortices. The vertical light blue line represents the distance A, defined as the vertical distance from the tibial joint line to the proximal tibial tubercle. The 3 pink lines represent the distances of 2A, 3A, and 4A from the tibial joint line. The midpoints of the pink lines are annotated with a green dot, which is used to determine the tibial shaft axis, in this case using the 2A and 3A lines. The dark blue lines represent the posterior tibial slope, defined as the angle between the tibial shaft axis and the medial tibia joint line. The bony landmarks are labeled as follows: 0, proximal patella; 1, distal patellar articular surface; 2, distal pole of patella; 3, anteriomedial proximal tibia; 4, posteromedial proximal tibia; 5, proximal tibial tubercle.

A disentangled keypoint regression (DEKR) algorithm to automatically measure the PTS was developed based on lateral knee radiographs obtained from patients in the Republic of Korea. The algorithm was first trained to recognize 6 bony landmarks, and the tibial shaft anatomic axis was determined relative to these landmarks, enabling PTS calculation using uncalibrated images. Briefly, this model first defined a vertical distance from the tibial joint line to the level of the proximal tibial tuberosity as distance “A” (Figure 1). Then, lines parallel to the joint line—connecting the anterior and posterior tibial cortices—were drawn at distances of 2A, 3A, and 4A from the tibial joint line. The midpoints of these 3 horizontal lines were then used to define 2 possible tibial shaft axes, representing short and long lateral knee radiographs. The short tibial axis was defined as a line connecting the midpoints of the 2A and 3A lines. The tibial joint line was annotated by connecting the anterior and posterior apexes of the medial tibial plateau.

The automated model was trained on long lateral radiographs of normal knees, osteoarthritic knees, and knees with implants (knee arthroplasty). When tested on lateral knee radiographs obtained in patients from the Republic of Korea, the model performed well, demonstrating perfect intrarater reliability of 1.00 for both long and short radiographs. The interrater reliabilities (compared with manual human measurements of the same knees) for the short and long radiograph models were found to be 0.91 and 0.92, respectively. Both models were also found to be significantly more time-efficient compared with manual PTS measurement.

### Data Source

Patients who had undergone ACL surgery at a single institution in Norway between 2006 and 2012 were eligible for inclusion. Besides ACL insufficiency, skeletal maturity was required for inclusion in the study. Additional inclusion criteria included an acceptable exposure (to allow a clear reading of bony landmarks) and at least 80% overlap of femoral condyles to ensure acceptable rotational alignment.^5^ As lateral knee radiographs captured at the Norwegian institution are uncalibrated, the previously defined ratio measurement based on the automated PTS model was used to ensure an acceptable minimum tibial length. First, 1A was defined as the vertical distance from the joint line to the level of the tibial tuberosity (see Figure 1 for an illustration of 1A distance). Images with a tibial length <3 times the 1A distance (3A) were excluded, as this was defined as the minimum tibial length required by the deep learning model.

### Tibial Slope Measurement

**Figure 2.**
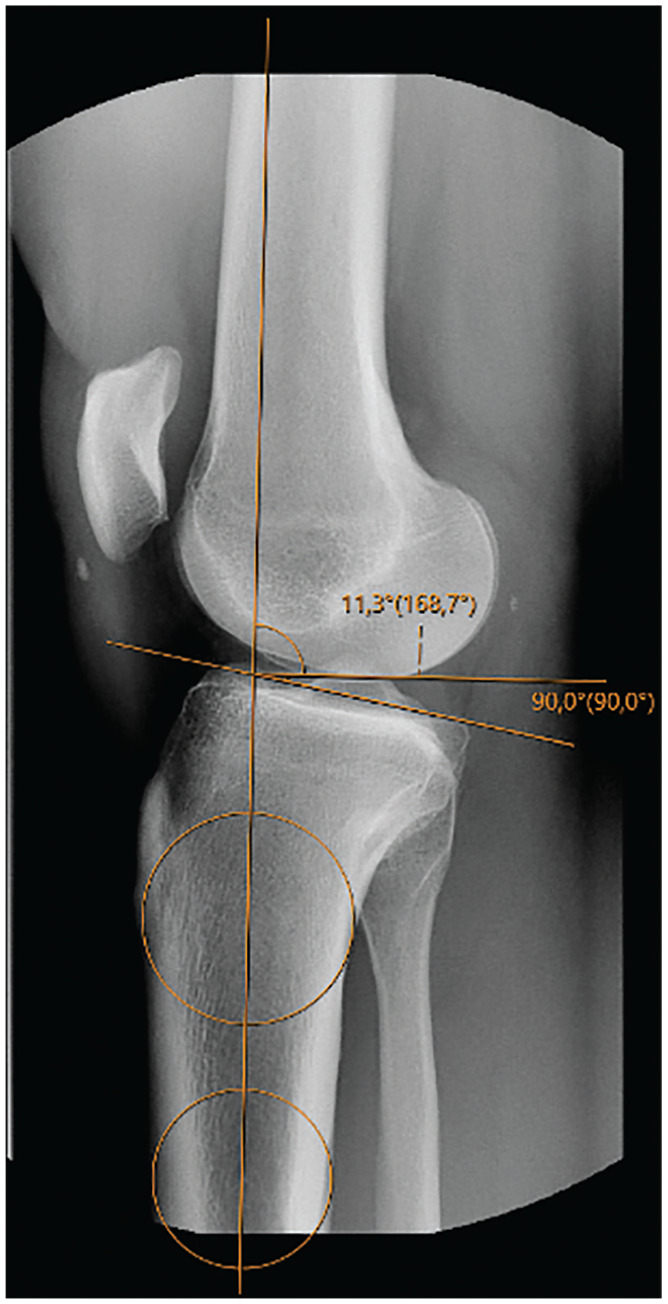
Manual measurement of the slope applying the 2-circle method, where the line through the 2 circles is a representation of the anatomic axis and the angle between a tangential line and line perpendicular to the medial tibial plateau was used as a representation of the tibial slope—in this case 11°.

Sagittal radiographs were acquired from the picture archiving and communications system and measured using IDS7 software (Sectra Medical) with a 2-circle method similar to that of Giffin et al.^4^ A proximal circle was placed at the level of the tibial tuberosity, and a distal circle was placed as far distal along the tibial shaft as images allowed. A line was drawn through the center of the circles as a representation of the tibial anatomic axis (Figure 2). The proximal tibial axis has formerly been shown to accurately represent the mechanical axis of the tibia on short lateral knee radiographs.^13,18^ The medial tibial plateau was outlined so that a tangential line could be drawn. The angle between the tangential line and a line perpendicular to the axis of the tibia was measured and used as a representation of the tibial slope. Finally, the time spent performing measurements was recorded for both the manual and automated methods.

Manual PTS measurements were calculated by 2 experienced orthopaedic clinicians. After initial concordance training between the examiners on a training set of radiographs not included in the study, readings were made by the 2 examiners to establish interrater reliability in manual PTS measurements. Because an acceptable manual interrater reliability was expected based on former studies applying similar methods,^3^ only 1 manual reader measurement was used in performing interrater reliability of automated versus manual measurements. A de-identified screenshot of the same images was then uploaded to an online interface of the deep learning model (Connecteve) to establish the interrater reliability between the computer model and the manual measurements. Measurements were then repeated 4 weeks later to determine the intrarater reliability. Outliers (defined as the 5% most extreme mean absolute differences) were visually inspected for systematic errors and omitted from the final part of analyses. This manual interpretation and exclusion of outliers was done to minimize the effect of clearly erroneous readings that would be obvious during use of the model, and the 5% cutoff was selected based on a previous model that demonstrated a 4.4% outlier rate.^9^

### Statistical Analysis

The mean and standard deviation, along with range, were calculated for demographic data of the included patients. For both the manual reading and automated reading of PTS, the intraclass correlation coefficient (ICC) was established for interrater and intrarater reliability, using the 2-way random absolute agreement in SPSS Version 25.0 (IBM Corp). Furthermore, a Bland-Altman plot was included to visually analyze the degree of agreement between manual and automated readings of slope.

## Results

**Figure 3.**
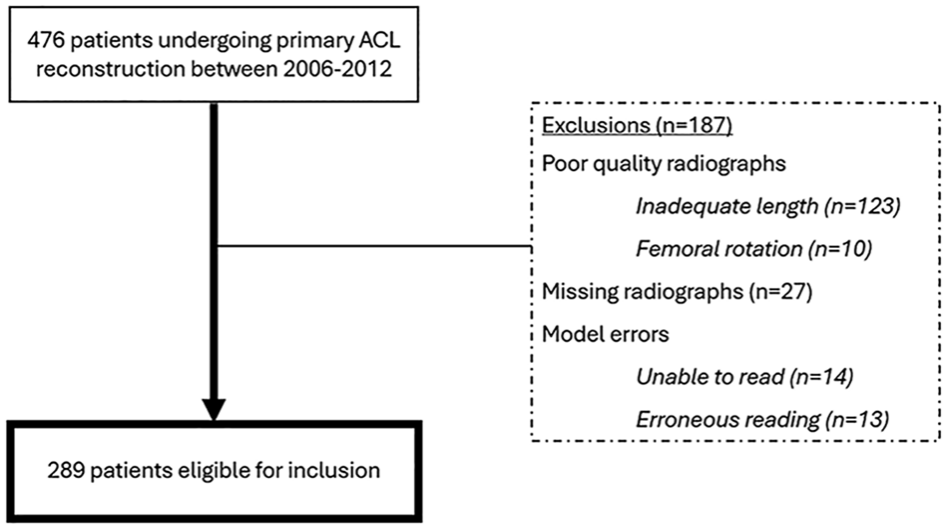
Flowchart for inclusion of images into the study. ACL, anterior cruciate ligament.

**Figure 4.**
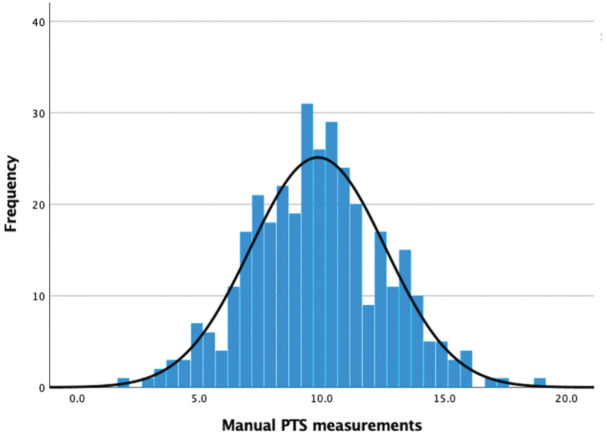
Distribution of manual slope measurements in the current cohort. PTS, posterior tibial slope.

Of 476 images screened for inclusion, 289 met the inclusion and exclusion criteria and were included into the study (Figure 3). Of these included images, 52.2% were from female patients, 51.2% were left knees, and the mean age at image acquisition was 29.7 years (SD, 11.2 years). From the manual measurements using the 2-circle method, a mean tibial slope of 9.7° (SD, 2.7°; range, 3.0°-19.1°) was found. For the automated reading using the short module, the mean tibial slope was 9.2° (SD, 3.4°; range, 0.2°-16.7°). An increased PTS, conventionally defined as >12°,^17^ was seen in 48 (16.6%) images based on the manual measurements and 62 (21.5%) images using the automated technique. In total, 28 (9.7%) were identified as having a PTS >12° by both the manual and the automated techniques. The distribution of measured slopes in the cohort is presented in Figure 4.

The interrater and intrarater reliabilities of the 2-circle method were found to have ICCs of 0.86 and 0.92, respectively, representing an excellent agreement between examiners.^2^ The mean absolute difference between the 2 readers was 0.17° (SD, 1.7°).

**Figure 5.**
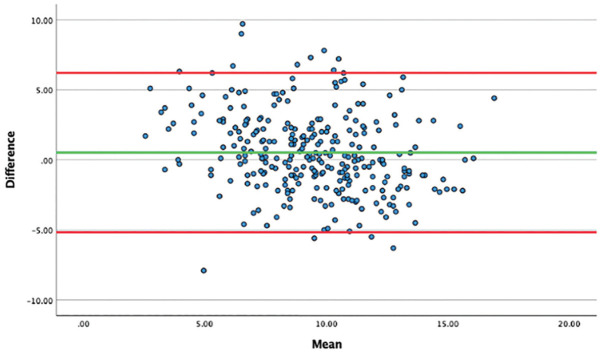
Bland-Altman plot displaying the mean differences between the manual measurements and automated model measurements.

When comparing the manual measurements with the automated model measurements for the entire data set (289 images), the ICC was 0.73. When outliers were removed, the ICC was 0.80 (272 patients). Intrarater reliability was a perfect 1.00 for the automated model. The mean absolute difference between the manual reading and the model was 0.52° (SD, 2.9°). When looking at potential systematic differences between measurement methods (Figure 5), there seems to be only a small positive bias toward higher manual readings. Overall, the mean time spent performing manual measurement was 52.5 seconds compared with 28.2 seconds spent on screenshotting, uploading, and acquiring automated measurements (*P* < .05).

A manual inspection of the 17 outliers (defined as the 5% most extreme mean absolute differences between manual and automated PTS measurements) revealed 2 short images that should have been excluded and 1 image with a grossly incorrectly annotated line along the tibial plateau. The remaining 14 images represent disparities in measurement due to inherent differences in the measurement methods. The model reported a PTS measurement that was less than the manual human measurement in 13 of the 15 discrepancies. The agreement between the model and manual measurement for these outliers was 0.02. To assess whether human error was the cause of the difference in the 5% most extreme outliers, the interrater reliability of the 2-circle model was established and found to have an ICC of 0.85.

## Discussion

The most significant finding of this study was that for the majority (95%) of the short lateral knee radiographs obtained in patients with an ACL deficiency in Norway, there was good agreement (ICC, 0.80) between the novel deep learning model and manual PTS measurement. Furthermore, the model demonstrated perfect intrarater reliability and improved time efficiency over manual measurement. However, for the 5% most divergent measurements, the level of agreement was atrocious, with an ICC of 0.02. While this study suggests optimism regarding the external validity of the automated model when tasked with measuring the PTS on short lateral knee radiographs, it also highlights the importance of further refinement and external validation to minimize the extreme outliers.

To our knowledge, this study represents the first effort to externally validate an automated method of calculating the PTS. This crucial step of external validation before implementation of novel artificial intelligence–based models into clinical practice is uncommonly performed.^11,15^ This step is important as it enables the assessment of model generalizability and may increase trust and acceptance regarding the utility of the model. Challenges limiting the feasibility of model external validation include the volume and suitability of data required, data transfer regulations, and the lack of a defined acceptable level of model performance, which typically decreases between the original model development and the external validation testing.^10,11^

There is inherent value in a valid and reliable PTS measurement technique, especially one that is automated and freely available. As the importance of the PTS has been increasingly reported, it is also apparent that several different PTS measurement techniques have been described and applied throughout the literature. This may lead to confusion or inappropriate application of described PTS cutoff values in clinical decision-making if the clinician is using a different measurement technique or inaccurately applying the described technique. In this sense, each researcher and clinician may be using a slightly different “yardstick” when assessing the PTS, especially when one considers the imperfect inter- and intrarater reliabilities of the individual techniques. We therefore propose a need for a standardized universally accepted technique that enables all researchers and clinicians to use the same yardstick when measuring, reporting, and clinically applying the PTS. This would ensure that all are speaking the same language when evaluating the PTS and its clinical relevance.

The model evaluated in this study has several attributes that support its suitability to act as a standardized PTS measurement tool. First, the ICC between the manual and automated PTS measurements was good for 95% of the evaluated images. This suggests validity of the model, which is especially valuable considering the images were noncalibrated short-leg radiographs, which are commonly obtained in clinical settings. Another advantage of this tool is that the intrarater reliability was perfect. This suggests that the same PTS will be produced each time the same image is analyzed, ensuring consistency when the same image is measured multiple times. It should be noted, however, that new images of the same patient may still produce varying PTS values given differences in tibial rotation or position between images. Finally, the measurement of PTS is quick and facile through the free online interface. De-identified images can be rapidly analyzed through manual image upload, drag-and-drop, or copy-and-paste mechanisms.

A similar tool for automatically measuring the PTS on lateral knee radiographs was previously developed by Lu et al.^9^ They created a deep learning U-net model using 300 postoperative short lateral knee radiographs obtained in patients who underwent ACLR. Subsequently, they internally validated the U-net model against human measurements using 90 independent pre- and postoperative images. They found a Dice similarity coefficient (DSC) of 0.885 for image segmentation, indicating good agreement between manual segmentation and that performed by the U-net model. Regarding PTS measurement, they reported an interrater reliability of 0.84 between the 2 manual reviewers and no significant difference between the manual and deep learning measurements. There is no report of the ICC between the deep learning model and the manual PTS measurement directly; however, the authors report that approximately 4.4% of the images were considered outliers, with >5° disagreement. In comparison, the internal validation of the DEKR algorithm used in this study showed an ICC of 0.91 for short images (2A/3A) and 0.92 for long images (2A/4A), representing excellent agreement between automated and manual PTS.

There are several differences in methodology between the study by Lu et al^9^ and the model evaluated in this study. First, the training data set for the DEKR algorithm consisted of 9227 images, compared with 300 images for the U-net model, while the validation test data set consisted of 230 images for the DEKR algorithm compared with 90 for the U-net model. The study by Lu et al also used a distance-based measurement of PTS, relying on calibrated images to determine the tibial shaft axis, defined as a line connecting the midpoints of the anterior and posterior tibial cortices 5 cm and 15 cm, respectively, below the joint line. In contrast, the model developed in the Republic of Korea uses a novel landmark-based method that is designed to evaluate the PTS on uncalibrated images that may be <15 cm of tibial length. The DSC value of 0.885 reported by Lu et al also represents the mean DSC between the U-net model and human measurements for segmentation of these areas (in contrast to PTS measurement), indicating further differences in methodology and statistical analysis between the 2 model development studies.

### Limitations

There are limitations to the present study. First, the validity of the automated PTS measurement tool was evaluated through comparison of its measurements with manual measurements obtained via a different technique. For this reason, imperfect agreement is expected because the tibial shaft axis determined using a different method would affect the PTS. The original model was also developed using knees that were normal, arthritic, or had previously undergone arthroplasty, whereas the external validation cohort consisted of ACL-deficient knees. That, combined with differences in imaging techniques and patient characteristics between the Republic of Korea and Norway, may also affect the accuracy of the automated PTS model when applied to this cohort. Nonetheless, the accuracy for 95% of the images was still considered good (ICC, 0.80). Furthermore, the interrater reliability between the 2 surgeons’ measurements was also good, with an ICC of 0.86. Another potential limitation is the fact that the images uploaded to the automated PTS measurement tool were screenshots of the original Digital Imaging and Communications in Medicine (DICOM) files rather than the original DICOM images themselves. This was done to eliminate the identifiable patient information from the radiographs before upload to the online interface but may have resulted in a degradation in image quality, potentially affecting the accuracy of the model.

The final and arguably most significant limitation relates to the fact that for 5% of the images evaluated, there was substantial disagreement between the automated model and manual measurements, with manual interpretation resulting in higher PTS measurements the majority of the time. Although this discordance represented a minority of the images, it could have substantial implications for those patients if used in a clinical setting. Blind overreliance on the automated model is therefore not recommended, and users should visually inspect the annotations and PTS calculations for accuracy if using this model in clinical and research settings. In addition, efforts to improve the model should be undertaken, with subsequent further external validation to assess model performance when applied to patients from different institutions.

## Conclusion

External validation of a novel landmark-based deep learning algorithm to automatically measure the PTS from short lateral knee radiographs demonstrated good agreement compared with manual measurement for 95% of the images. The agreement was poor for 5% of the evaluated images. The model was also reliable, with perfect intrarater reliability on repeat evaluation of the same images, and required less time than manual PTS measurement. This suggests the overall validity and reliability of the model, although further efforts should be made to improve the model and minimize outliers.
